# Smoking, Alcohol Consumption, and Atrial Fibrillation: Mendelian Randomization Study

**DOI:** 10.1007/s12012-025-09964-8

**Published:** 2025-02-22

**Authors:** Xuejiao Ye, Qian Wu, Qianyu Lv, Xinzheng Hou, Yingtian Yang, Chenyan Yang, Shihan Wang

**Affiliations:** Department of Cardiovascular Diseases, Guang’anmen Hospital, China Academy of Chinese Medical Sciences, No. 5 Beixiange, Xicheng District, Beijing, China

**Keywords:** Smoking, Active smoking, Secondhand smoke exposure, Alcohol consumption, Atrial fibrillation, Mendelian randomization

## Abstract

Smoking, secondhand smoke exposure, and alcohol consumption are significant risk factors that contribute to an increased global burden of cardiovascular diseases. However, the casual relationship between smoking, passive smoking, alcohol consumption, and atrial fibrillation (AF) remains uncertain. Conventional observational studies are difficult to draw conclusion on high-quality causality. To elucidate the association between smoking, secondhand smoke exposure, alcohol consumption, and AF, we conducted this two-sample Mendelian randomization (MR) analysis. Smoking encompasses current tobacco smoking, ever-smoked, and light smokers, with light smokers being defined as at least 100 smokes in lifetime, as well as secondhand smoke exposure, which is characterized by workplace had a lot of cigarette smoke from other people smoking: Often. Alcohol consumption encompasses diagnoses—secondary ICD10: Z72.1 Alcohol use and the frequency of alcohol intake. Genetic variants associated with smoking and alcohol consumption were obtained from the IEU Open GWAS project and subsequently selected as instrumental variables (IVs). The corresponding variants associated with AF were also retrieved from the IEU Open GWAS project. The primary MR method utilized was the inverse-variance weighted (IVW). To assess the robustness of our results, multiple supplementary methods were utilized, including the weighted median (WM), MR-Egger regression, MR-PRESSO, MR-Egger intercept test, and the leave-one-out method. A reverse MR analysis was also conducted to determine the potential existence of reverse causality. Genetic predictions indicate a causal relationship between active smoking (current tobacco smoking, *P*_*-val*_ = 0.019, OR: 1.413, 95% CI = 1.058–1.888; ever smoked, *P*_*-val*_ = 0.049, OR: 1.355, 95% CI = 1.001–1.834; light smokers, *P*_*-val*_ = 0.001, OR: 1.444, 95% CI = 1.154–1.806) and AF. No causal association was found between secondhand smoke exposure, alcohol consumption phenotypes, and AF. Additionally, the reverse MR analysis did not reveal any evidence of reverse causality from AF to active smoking. This study provides MR evidence supporting a causal association between active smoking and AF. The significance of smoking cessation is underscored by its potential to prevent or mitigate the risk of AF. Furthermore, the impact of secondhand smoke exposure and alcohol consumption on AF, as well as the causality among these factors, warrants further investigation.

## Introduction

Atrial fibrillation (AF) is the most common rapid arrhythmia in clinical practice. According to epidemiological studies, there are currently over 33.5 million AF patients worldwide, and the number of people with AF is growing by 5 million annually [12]. Patients with AF may experience palpitations, dizziness, dyspnea, and darkness due to hemodynamic alternations induced by the irregular and rapid contractions of the atrial muscles. The occurrence of frequent AF episodes substantially increases the risk of severe cardiovascular and cerebrovascular events, such as stroke, heart failure, and thromboembolism. Owing to the rapid aging of the global population, the prevalence of AF is predicted to reach a new peak by 2050, accompanied by a continuous escalation of the associated socioeconomic burden [41].

AF can either initiate or be a consequence of the progression of cardiovascular disease [24]. Although the pathogenesis of AF is still not fully understood, it has been determined that at least 56% of cases can be attributed to identifiable risk factors [22]. These encompass both underlying conditions, such as coronary heart disease (CHD), hypertension, hyperthyroidism, and obstructive sleep apnea (OSA), and modifiable risk factors like obesity, smoking, alcohol consumption, and a sedentary lifestyle. The detrimental effect of smoking and alcohol consumption on AF has been well documented in clinical practice. Nevertheless, prior research, which was mainly observational studies or randomized controlled trials (RCTs), has been susceptible to biases and confounding variables. Consequently, a definitive causal relationship between smoking, secondhand smoke exposure, alcohol consumption, and AF remains to be elucidated [10, 19, 38, 42, 60].

To our knowledge, establishing causality is pivotal in epidemiological research. However, both observational studies and RCTs are susceptible to the influences of confounding factors and reverse causality, which may undermine the reliability of deduced causal relationships. Genetic variation occurs prior to the onset of disease and remains unaffected by postnatal lifestyle and environmental factors [26, 34]. MR analysis, a genetic epidemiological technique [27], utilizes genetic variations to assess the effect of exposure on disease, thereby alleviating the impact of potential confounding factors and reverse causality [53]. Therefore, we conducted this bidirectional MR analysis to assess the causality between active smoking, passive smoking, alcohol consumption, and AF. Our hypothesis posits a causal relationship between active smoking, passive smoking, alcohol consumption, and AF.

## Methods

### Study Design

**Fig. 1:**
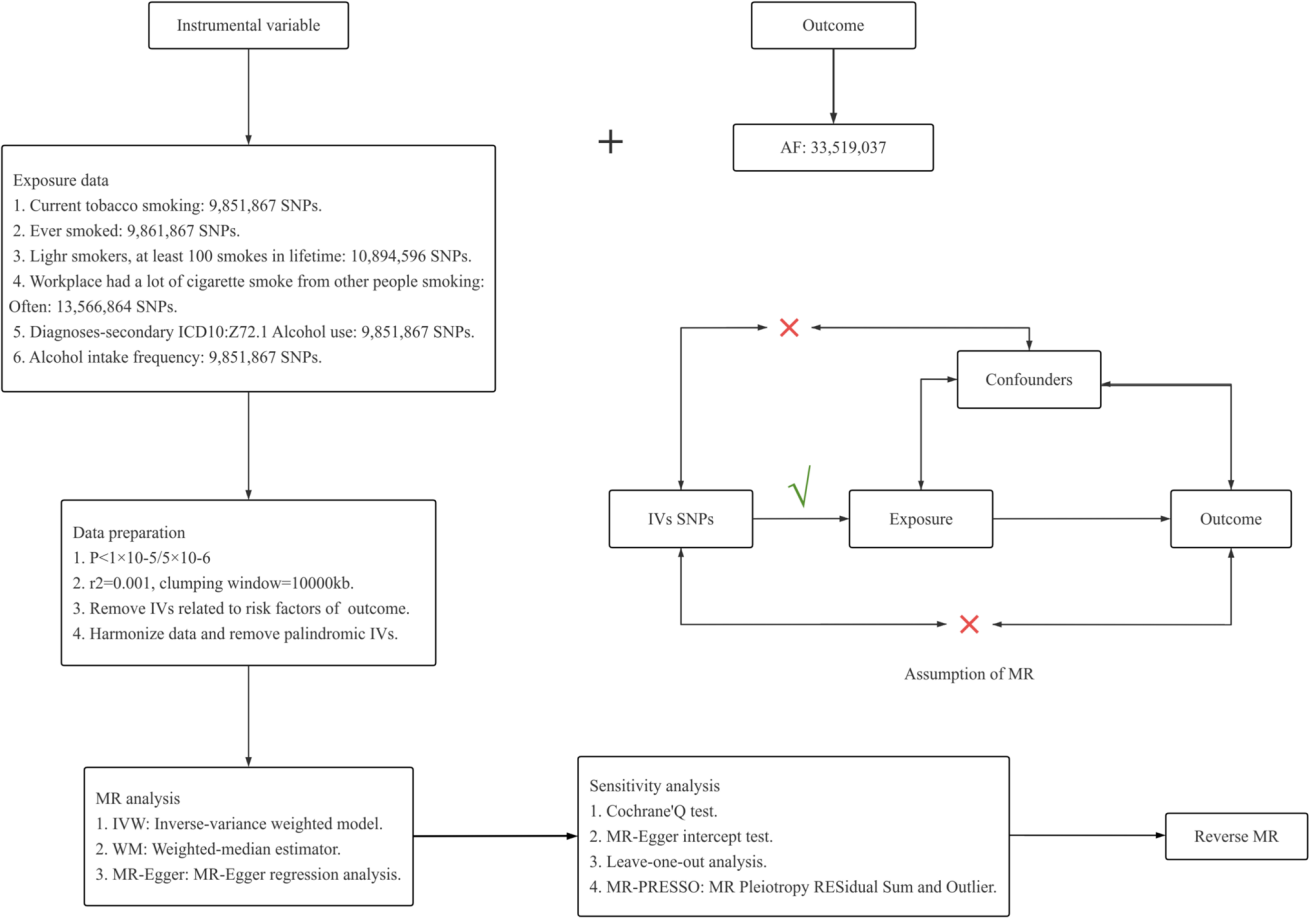
Overview of the study design and major assumptions of this MR study. SNPs, single-nucleotide polymorphism; AF, atrial fibrillation; IVs, instrumental variances

This two-sample MR study aimed to investigate the causal relationship between smoking, alcohol consumption, and AF. Figure 1 presents an overview of the MR analysis and its underlying assumptions. To ensure the reliability of the results, three essential hypotheses need to be met (as presented in Fig. 1): (1) Genetic variants should be robustly correlated with the exposure (relevance); (2) genetic variants should have no association with confounding factors (exchangeability); and (3) genetic variants should affect the outcome only through exposure (exclusion restriction), excluding any other potential pathways, that is, horizontal pleiotropy is unacceptable. Only genetic variants that meet these criteria are qualified to serve as instrumental variables (IVs) in MR analysis [3]. This MR study was reported following the Strengthening the Reporting of Observational Studies in Epidemiology Using Mendelian Randomization (STROBE-MR) guidelines [51].

### Data Sources and Selection of Instrumental Variables (IVs)

In this MR analysis, the smoking phenotypes included active smoking and exposure to secondhand smoke. Active smoking was categorized according to smoking patterns and quantities, including current tobacco smoking, ever smoked, and light smokers, with light smokers being defined as individuals who have smoked at least 100 smokes in lifetime. Secondhand smoke exposure refers to workplace had a lot of cigarette smoke from other people smoking: Often. Alcohol consumption phenotypes include diagnoses—secondary ICD10: Z72.1 Alcohol use and the frequency of alcohol intake. Summary statistics for smoking, alcohol consumption, and AF were obtained from the MRC-IEU [IEU OpenGWAS project (https://mrcieu.ac.uk)]. The detailed baseline characteristics of the sample, including the sample size for exposure and outcome, the group sizes for experimental and control arms, the number of single-nucleotide polymorphism (SNPs), ethnicity, gender, and data source, are presented in Table 1. All of the included samples were European ancestry. To ensure the quality of IVs, we conducted rigorous quality control procedures on single SNPs: (1) Considering the relatively small number of SNPs identified at the genome-wide significance threshold (*P* < 5e−08), which might lead to the omission of potentially significant results [46], we adopted the locus-wide significance threshold (*P* < 5e−06 [25] and *P* < 1e−05 [41]), a criterion commonly applied in MR studies; (2) SNPs in linkage disequilibrium (defined as *r*^2^ = 0.001, Clumping distance = 10000 kb); (3) palindromes and incompatible SNPs were removed during the harmonization of exposure and outcome statistics, as were SNPs related to exposure that could not be matched in the GWAS outcome statistics. The F-statistics for each SNP associated with exposure exceeded the empirical threshold of 10, indicating that the SNPs utilized had sufficient strength [59].

$$ F = \frac{N - k - 1}{k} \times \frac{{N \times R^{2} }}{{1 - R^{2} }} $$

(*N* = sample size of the exposure, *k* = the number of selected SNPs, and *R*^2^ represents the phenotype variance induced by the SNPs.) When *R*^2^ is not available, we used the formula:

$$ R^{2} = 2 \times MAF \times 1 - MAF \times \beta /{\text{SD}}^{2} $$

(*β* = the effect value of the genetic variant of the exposure, MAF = the effect allele frequency of selected SNPs, SD = *SE* × $\sqrt{N}$, *SE* = the standard error of the genetic variant of the exposure, *N* = sample size of the exposure.)

**Table 1:** Baseline characteristics for the instrumental variables

Trait	Sample size (*n*)	Case (*n*)	Control (*n*)	nSNP	Ethnicity	Gender	Data source
AF	1,020,836	60,620	970,216	33,519,037	European	Males/females	PMID:30061737
Current tobacco smoking	462,434	NA	NA	9,851,867	European	Males/females	MRC-IEU
Ever smoked	461,066	280,508	180.558	9,861,867	European	Males/females	MRC-IEU
Light smokers, at least 100 smokes in lifetime	90,517	41,027	49,490	10,894,596	European	Males/females	Neale Lab
Workplace had a lot of cigarette smoke from other people smoking: Often	89,803	14,941	74,862	13,566,864	European	Males/females	Neale lab
Diagnoses—secondary ICD10: Z72.1 Alcohol use	463,010	3,685	459,325	9,851,867	European	Males/females	MRC-IEU
Alcohol intake frequency	462,346	NA	NA	9,851,867	European	Males/females	MRC-IEU

### Statistical Analysis

#### MR Analysis

We utilized the two-sample MR to assess the relationship among smoking phenotypes, alcohol phenotypes, and AF. Multiple genetic variants were utilized as IVs to avoid allele scoring, thereby enabling the exploration of key hypotheses, the assessment of pleiotropy, and the efficient execution of sensitivity analyses [14]. The inverse-variance weighted (IVW) was used as the primary analysis method, providing the most accurate and robust estimates when all genetic variants served as valid instruments [6]. Complementary to the IVW method, other methods like the weighted median (WM) and MR-Egger regression [4] provide wider confidence intervals (CI) [5, 52]. The WM method yields a reliable estimate provided that at least 50% of the instruments are valid. Whereas the MR-Egger method is adept at testing causal estimates when the majority of genetic variants display horizontally pleiotropic (> 50%) [4]. In the IVW analysis, the MR-PRESSO method was further utilized to detect bias resulting from outliers due to horizontal pleiotropy and to eliminate anomalous SNPs, thereby enhancing the reliability of causal estimates [57]. A *P*-value of less than 0.05 was deemed statistically significant [33]. In this exploratory study, we did not perform an adjustment for multiple testing [58].

#### Sensitivity Analysis

The MR-Egger intercept test was utilized to assess the existence of directional pleiotropy, as indicated by its intercept term [7]. The MR-PRESSO method also detects directional pleiotropy by identifying potential outliers and subsequently recalculating estimates after their exclusion. Cochrane’s *Q* statistic was calculated for the IVW method to quantify the heterogeneity among the IVs, with *P*-value greater than 0.05 indicates the absence of significant heterogeneity, in which case the fixed-effects IVW model provides a more conservative estimate. Otherwise, the random-effects IVW model is utilized [17]. Palindromic variants were removed during the harmonization of summary statistics. We further implemented a leave-one-out approach to determine whether any single SNP could induce bias in the summary estimates. Finally, a reverse MR analysis was conducted using the same procedures to investigate potential reverse causality. If the causality between smoking, passive smoking, alcohol consumption and AF is deemed significant by two or more MR methods, we regard the result was robust [37]. All MR analyses were conducted using the packages “Two SampleMR 0.5.11,” “MendelianRandomization,” and “MR-PRESSO” in R software (version 4.3.2).

## Results

### Basic Characteristics of IVs Related to Exposures and Outcome

IVs were selected in accordance with the previously described procedure. Genetic variants associated with both exposure and outcome were identified from the following sources: (1) IEU public GWAS data repositories; (2) the Neale Laboratory of Statistical Genetics and Data Science; and (3) the publication with the identifier PMID:30061737. The GWAS statistics for current tobacco smoking contains 101 loci, with a sample size of 462,434. For the ever-smoked status, there were 123 loci from 461,066 samples. For light smokers (defined as at least 100 smokers in lifetime), 24 loci were identified from 90,517 samples, and for secondhand smoke exposure, 16 loci were obtained from 89,803 samples. The diagnostic criteria for alcohol use were based on the International Classification of Diseases, 10th Revision (ICD10) codes, with GWAS statistics including 11 loci from 463,010 samples for alcohol use, and 76 loci from 462,346 samples for alcohol intake frequency. Comprehensive details of the GWAS statistics are presented in Table 1.

### Effect of Smoking Phenotypes on AF

**Fig. 2:**
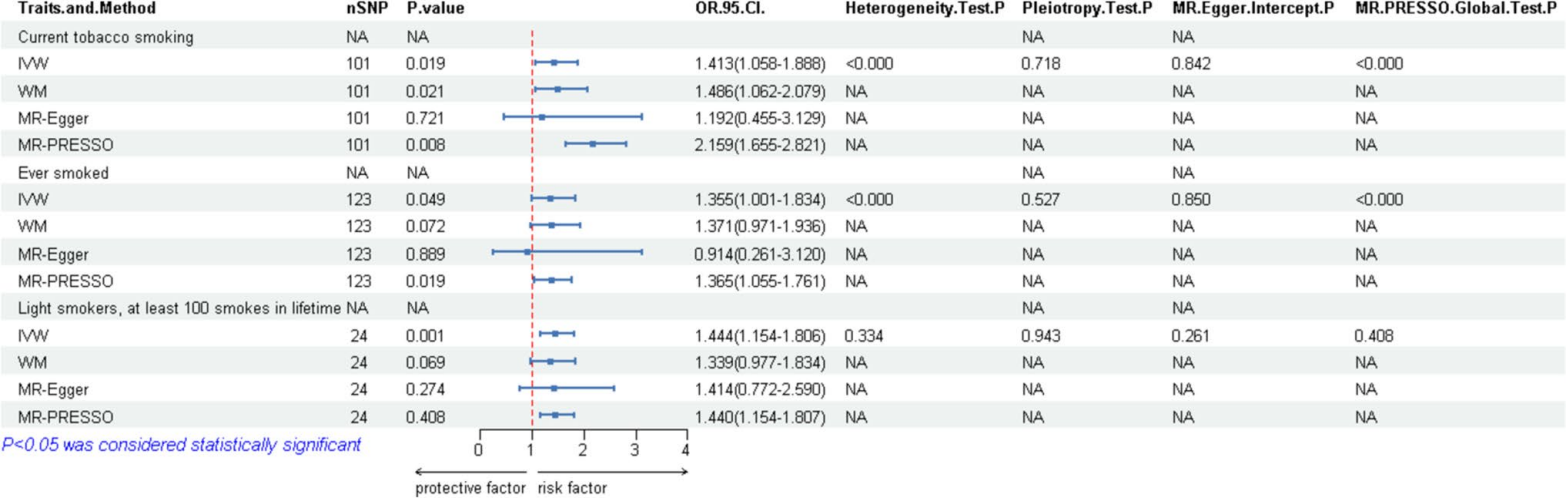
MR results of smoking with significance causal relationships to AF

**Fig. 3:**
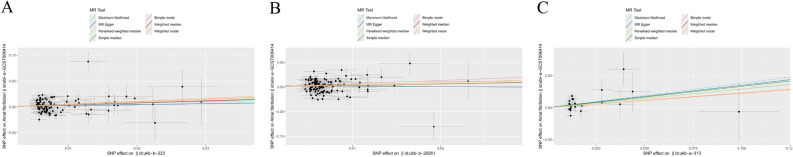
Scatter plot of the causal association between smoking phenotypes and AF. **A** Scatter plot for current tobacco smoking and AF; **B** Scatter plot for ever smoked and AF; **C** Scatter plot for light smokers: at least 100 smokes in lifetime and AF

**Fig. 4:**
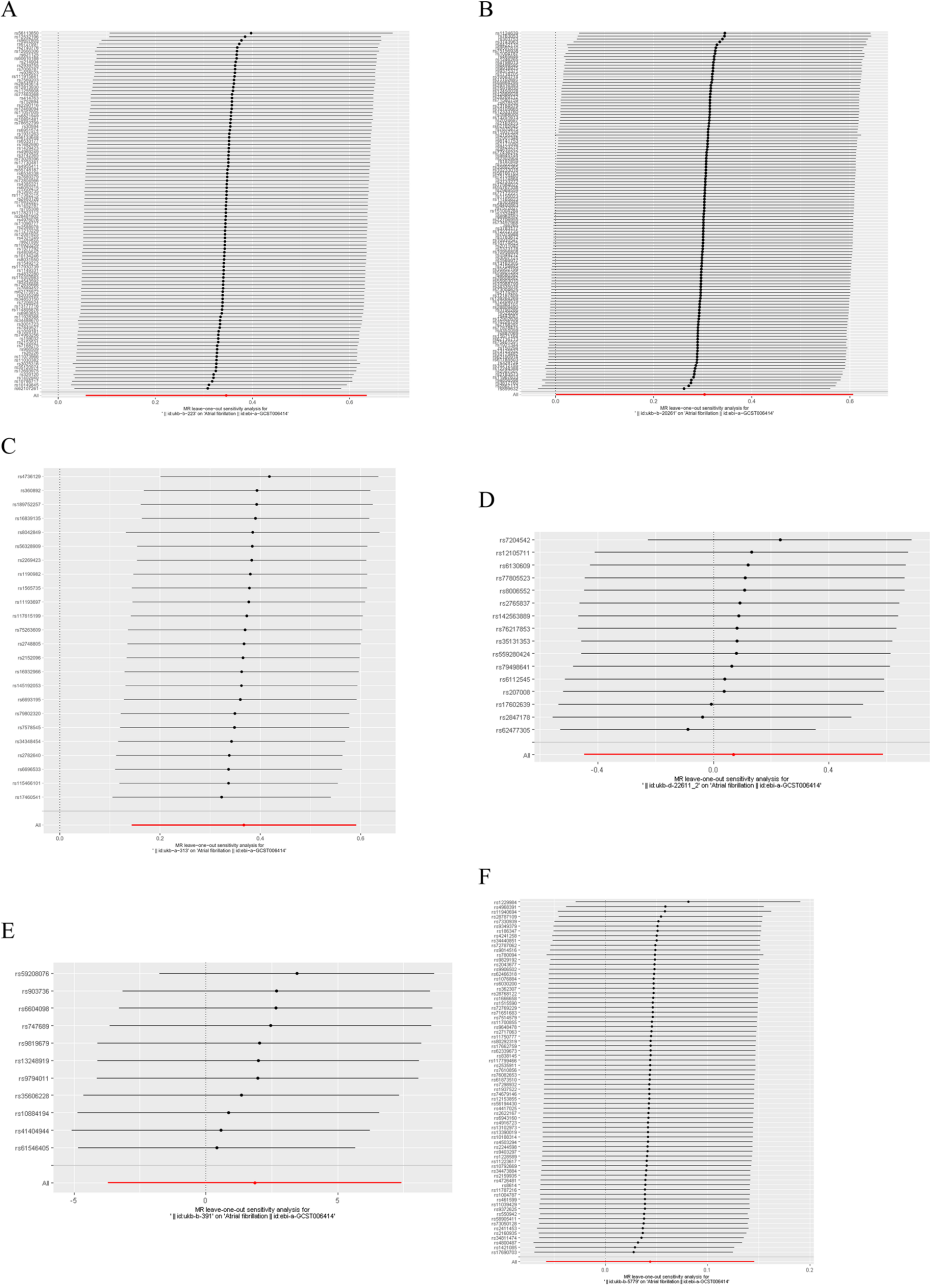
Leave-one-out analysis of the causal association between smoking phenotypes, alcohol phenotypes, and AF. **A** Leave-one-out analysis for current tobacco smoking and AF; **B** Leave-one-out analysis for ever smoked and AF; **C** Leave-one-out analysis for light smokers: at least 100 smokes in lifetime and AF; **D** Leave-one-out analysis for secondhand smoke exposure and AF; **E** Leave-one-out analysis for diagnoses—secondary ICD10: Z72.1 Alcohol use and AF; F. Leave-one-out analysis for alcohol intake frequency and AF

A total of 101 independent single-nucleotide polymorphisms (SNPs) were designated as genetic IVs for current tobacco smoking, 123 for ever-smoked status, 24 for light smokers, and 16 for secondhand smoking. The F-statistics for all SNPs exceeded 10, as presented in Table 2. In our MR analysis, current tobacco smoking (OR = 1.413, 95% CI: 1.058–1.888, *P* = 0.019), ever-smoked status (OR = 1.355, 95% CI: 1.001–1.834, *P* = 0.049), and light smokers (OR = 1.444, 95% CI: 1.154–1.806; *P* = 0.001) were associated with an increased risk of AF, as depicted in Fig. 2 and Table 2. However, no causal relationship was determined between secondhand smoke exposure and AF (OR = 1.073, 95% CI: 0.639–1.801, *P* = 0.789). Cochrane’s *Q* test demonstrated the absence of heterogeneity in light smokers (*P* = 0.334), and secondhand smoke exposure (*P* = 0.100), yet it revealed heterogeneity in current tobacco smoking (*P* < 0.001) and ever-smoked status (*P* < 0.001). We utilized the IVW random-effects model to address heterogeneity and ensure the reliability of our results. The heterogeneity might be attributed to diverse genetic backgrounds, the complexity of smoking behavior, and potential confounding factors. To further investigate the sources of heterogeneity, we conducted MR-PRESSO analysis to detect and correct potential horizontal pleiotropy and outliers. MR-PRESSO analysis detected anomalous SNPs that could violate the MR hypothesis and corrected them to reduce bias in MR analysis. The analysis revealed potential outliers in current smoking and ever smoked. After their removal, the corrected *P*-values were 0.021 and 0.017, respectively. The *P*-values of the Distortion test was all great than 0.05 (current smoking: *P* = 0.76, ever smoked: *P* = 0.899), indicating that the adjusted results remained stable and that our MR study findings were not substantially affected by outliers. In sensitivity analyses, no evidence of horizontal pleiotropy was observed in the pleiotropy test (current tobacco smoking, *P* = 0.718; ever smoked, *P* = 0.527; light smokers, *P* = 0.943; workplace exposure to secondhand smoking, *P* = 0.196, Table 2) and MR-Egger intercept analysis (current tobacco smoking, *P* = 0.842; ever smoked, *P* = 0.850; light smokers, *P* = 0.261; workplace exposure to secondhand smoking, *P* = 0.177) across all smoking phenotypes (Fig. 3). MR-PRESSO analyses identified between 1 and 6 outliers; nevertheless, the stability of the overall results was maintained after the removal of outliers, as presented in Table 2. We also performed leave-one-out sensitivity analysis, which further confirmed the robustness of our results for current tobacco smoking and light smokers. Nevertheless, the leave-one-out plot for ever-smoked and secondhand smoke exposure suggested potential influential SNPs driving the causal link between them and AF, indicating that these findings should be interpreted with caution, as presented in Fig. 4.

**Table 2:** MR analysis results of causal links between smoke phenotypes and AF

Exposure	nSNP	Method	OR (95% CI)	*P*-val	*P*-val. heterogeneity	*P*-val. pleiotropy	*P*	*F*
Current tobacco smoking	101	IVW	1.413 (1.058–1.888)	0.019	< 0.001	0.718	5e−06	30.02
		WM	1.533 (1.094–1.978)	0.013				
		MR-Egger	1.192 (0.455–3.129)	0.721				
		MR-PRESSO	2.159 (1.655–2.821)	0.008				
		MR-Egger intercept		0.842				
Ever smoked	123	IVW	1.355 (1.001–1.834)	0.049	< 0.001	0.527	5e−06	33.59
		WM	1.392 (0.993–1.930)	0.05				
		MR-Egger	0.914 (0.261–3.120)	0.889				
		MR-PRESSO	1.365 (1.055–1.761)	0.019				
		MR-Egger intercept		0.850				
Workplace had a lot of cigarette smoke from other people smoking: Often	16	IVW	1.073 (0.639–1.801)	0.789	0.100	0.196	5e−06	23.23
		WM	0.855 (0.461–1.000)	0.621				
		MR-Egger	2.330 (0.683–7.958)	0.198				
		MR-PRESSO	1.073 (0.640–1.801)	0.109				
		MR-Egger intercept		0.177				
Light smokers, at least 100 smokes in lifetime	24	IVW	1.444 (1.154–1.806)	0.001	0.334	0.943	5e−06	29.26
		WM	1.342 (0.966–1.854)	0.079				
		MR-Egger	1.414 (0.772–2.590)	0.274				
		MR-PRESSO	1.44 (1.154–1.807)	0.408				
		MR-Egger intercept		0.261

### Effect of Alcohol Consumption Phenotypes on AF

A total of 11 independent single-nucleotide polymorphisms (SNPs) were designated as genetic IVs for alcohol use, 76 SNPs were selected for alcohol intake frequency. The F-statistics for all SNPs exceeded 10, as presented in Table 3. Genetic predictions indicated no causal associations between alcohol consumption phenotypes and AF, including the diagnoses—secondary ICD10: Z72.1 Alcohol use (OR = 1.298, 95% CI: 0.002–1083.222, *P* = 0.940), and alcohol intake frequency (OR = 1.045, 95% CI: 0.944–1.157, *P* = 0.397). Cochrane’s *Q* test revealed no evidence of heterogeneity for diagnoses—secondary ICD10: Z72.1: Alcohol use (*P* = 0.135), yet it detected heterogeneity in alcohol intake frequency (*P* = 1.361e−8). We utilized the IVW random-effects model to address heterogeneity and ensure the reliability of our IVW analysis results. In sensitivity analyses, no horizontal pleiotropy was detected in the two alcohol consumption phenotypes through the pleiotropy test (diagnoses—secondary ICD10: Z72.1: Alcohol use:* P* = 0.938, alcohol intake frequency: *P* = 0.130) and the MR-Egger intercept analysis (diagnoses—secondary ICD10: Z72.1: Alcohol use:* P* = 0.984, alcohol intake frequency: *P* = 0.304). All the results are presented in Table 3. MR-PRESSO analyses identified 2 outliers; nevertheless, the stability of the overall results was maintained after the removal of these outliers, as shown in Table 3. We additionally performed a leave-one-out sensitivity analysis, which indicating the potential influence of specific SNPs on the causal relationship among diagnoses—secondary ICD10: Z72.1: Alcohol use, alcohol intake frequency, ever-smoked status, secondhand smoke exposure, and AF. Consequently, the present findings should be interpreted with caution, as shown in Fig. 4.

**Table 3:** MR analysis results of causal links between alcohol consumption phenotypes and AF

Exposure	Traits	nSNP	Method	OR (95% CI)	*P*-val	*P*-val. heterogeneity	*P*-val. pleiotropy	*F*	*P*
Diagnoses—secondary ICD10: Z72.1 Alcohol use	AF	11	IVW	1.298 (0.002–1083.111)	0.940	0.135	0.938	22.66	1e−05
			WM	1.264 (1.197e−03–1.335e+03)	0.947				
			MR-Egger	0.530 (1.338e−27–2.101e+26)	0.984				
			MR-PRESSO	2.866 (0.011–757.570)	0.136				
			MR-Egger intercept		0.984				
Alcohol intake frequency	AF	76	IVW	1.045 (0.944–1.157)	0.397	1.361e−8	0.130	56.62	5e−08
			WM	0.982 (0.873–1.152)	0.763				
			MR-Egger	0.893 (0.713–1.118)	0.327				
			MR-PRESSO	1.030 (0.947–1.121)	0.490				
			MR-Egger intercept		0.304

### Reverse MR Analysis

Ultimately, we conducted a reverse MR analysis on the outcomes that attained nominal significance, with the aim of ascertaining whether genetically predicted AF is causally associated with active smoking phenotypes. The procedures for the reverse MR analysis were consistent with those previously described. The reverse MR analysis did not report significant results, as presented in Table 4 (current tobacco smoking, OR = 1.001, 95% CI: 0.996–1.007, *P* = 586; ever smoked, OR = 0.998, 95% CI: 0.993–1.002, *P* = 0.279; light smokers, OR = 1.001, 95% CI: 0.992–1.009, *P* = 845).

**Table 4:** Reverse MR analysis results of causal links between smoke phenotypes and AF

Outcome	nSNP	Method	OR (95% CI)	*P*-val	*P*-val. heterogeneity	*P*-val. pleiotropy	*P*	*F*
Current tobacco smoking	74	IVW	1.001 (0.996–1.007)	0.586	< 0.001	0.045	5e−08	51.54
		WM	1.004 (0.997–1.010)	0.260				
		MR-Egger	1.010 (1.000–1.02)	0.045				
		MR-PRESSO	1.002 (0.997–1.007)	0.498				
Ever smoked	74	IVW	0.998 (0.993–1.002)	0.279	0.002	0.456	5e−08	51.54
		WM	0.999 (0.993–1.005)	0.693				
		MR-Egger	1.000 (0.992–1.009)	0.925				
		MR-PRESSO	0.998 (0.994–1.002)	0.372				
Light smokers, at least 100 smokes in lifetime	74	IVW	1.001 (0.992–1.009)	0.845	0.118	0.557	5e−08	51.54
		WM	1.002 (0.987–1.016)	0.818				
		MR-Egger	1.005 (0.988–1.023)	0.546				
		MR-PRESSO	1.001 (0.992–1.009)	0.873				

## Discussion

Numerous epidemiological studies have suggested that smoking and alcohol consumption are risk factors for various cardiovascular diseases. However, the direct causality between these factors and AF remains unknown. Conventional observational studies and RCTs have inherent methodological limitations, such as susceptibility to confounding factors and reverse causality, which MR studies can potentially overcome. In this study, we utilized a bidirectional MR approach to preliminarily assess the causal relationship between smoking phenotypes, alcohol consumption phenotypes, and AF. The primary finding of this research is that active smoking phenotypes are associated with an increased risk of AF. Specifically, current tobacco smoking potentially increases the risk of AF by 41.3% (OR = 1.413, 95% CI: 1.058–1.888, *P* = 0.019).

### The Association Between Smoking and AF

Large clinical trials and meta-analyses have demonstrated that tobacco constituents pose significant health risks, primarily affecting the heart, blood vessels, and lungs. Prior observational studies have identified a dose-dependent relationship between smoking and AF, with increased AF incidence associated with longer smoking duration and greater cumulative smoking exposure [1]. The Rotterdam study [21] reported a 51% and 49% increased risk of AF onset in current and former smokers, respectively, compared to non-smokers. The Atherosclerosis risk in Communities (ARIC) [9] demonstrated that smoking increases the risk of AF by 58% in European and American populations. Single-center studies conducted in China [31] and Japan [56] have also reached similar conclusions, suggesting that smoking is an independent risk factor for elderly patients with paroxysmal AF and those with new-onset AF. The American Heart Association (AHA), in its 2020 consensus statement, expanded the classic three-level management plan for AF to include lifestyle and risk factor management [13]. Collectively, these studies emphasize smoking as an independent risk factor for the incidence of AF, a relationship further substantiated by our research, which confirmed the causal association between active smoking and AF.

AF emerges as a consequence of the combined effects of multiple risk factors. Current research suggests that tobacco may promote the development of AF substrates and trigger AF through mechanisms like inflammation and oxidative stress. Firstly, studies have demonstrated that the cardiac effects of smoking are similar to the atrial matrix alternations requisite for the onset of AF. Animal models have revealed that nicotine can induce myocardial fibrosis and enhance atrial collagen expression, thereby increasing the risk of AF by approximately 8–15 times [48]. In vitro studies have further confirmed that nicotine can suppress the expression of miR-133 and miR-590 in fibroblasts, resulting in atrial fibrosis, atrial structural remodeling, and the perpetuation of AF [49]. Secondly, cardiac electrical activity is regulated by the interaction of sympathetic and parasympathetic nervous systems. Chronic heavy smoking can diminish vagal tone, attenuate the inhibitory effect on the sympathetic nervous system, disrupt atrial electrical stability, and subsequently increase the risk of AF. Additionally, tobacco’s harmful constituents can affect the stability of ion channels in cardiomyocytes [20]. Nicotine can block inward rectifying potassium channels, prolong the action potential of cardiomyocytes, and increase susceptibility to AF by disrupting the stability of myocardial ion channels [40, 49, 54].

Nicotine and tar can impair the vascular endothelium, provoke oxidative stress and inflammatory responses, and increase the risk of ischemia cardiovascular diseases, such as hypertension, CHD and heart failure, all of which are recognized risk factors for AF [11]. Clinically, patients with cardiovascular diseases like CHD, hypertension, and heart failure exhibit an increased risk of developing AF, potentially due to alternations in atrial homeostasis. Observational studies have indicated that the prevalence of AF among patients with CHD ranges from 6 to 21% [32]. In hypertensive patients, poor blood pressure control is substantially correlated with the recurrence of AF following ablation [2]. After adjusting for CHD and heart failure at baseline, the ARIC study [9] found a diminished association between smoking and AF. Compared to its effects on the heart, smoking has a more pronounced impact on the lungs. A large cohort study conducted in South Korea reported that reduced lung function is an independent risk factor for AF. Patients with obstructive or restrictive pulmonary dysfunction encounter a 42% and 49% higher risk of AF, respectively, in contrast to those with normal lung function [29]. The ARIC study also documented a negative correlation between obstructive pulmonary dysfunction and the risk of AF [30]. In summary, it is posited that smoking can indirectly increase the risk of AF through the induction of cardiopulmonary diseases. While the precise mechanisms through which pulmonary dysfunction contributes to AF remain elusive, it is hypothesized that they may involve hypoxia, inflammatory responses, and sympathetic nervous system activation. Consequently, smoking is unequivocally a substantial risk factor for the onset of AF, and ceasing smoking or maintaining non-smoking status is of paramount significance in mitigating the incidence of AF.

It is widely recognized that smoking exerts a detrimental impact not only on the health of smokers but also on that of individuals exposed to secondhand smoke. Numerous clinical trials have investigated the association between secondhand smoke exposure and the occurrence of AF, yet the results have been inconsistent. A case–control study conducted in Israel [44] indicated that women with long-term exposure to secondhand smoke had a 3.81fold increased risk of developing AF, and this risk was positively correlated with cumulative exposure duration. Groh et al. [8] identified that the incidence of AF exhibits a familial clustering pattern, and that prolonged exposure to secondhand smoke during childhood can increase the risk of AF by 18% in adulthood. Additional studies have demonstrated that individuals with extensive secondhand smoke exposure during fetal development or childhood are substantially more likely to develop AF in adulthood [15]. Collectively, these findings suggest that chronic secondhand smoke exposure may increase the risk of AF. Nevertheless, in our MR study, no genetic susceptibility was detected between secondhand smoke exposure and AF. Further research with larger cohorts and longer follow-up periods is warranted to elucidate the relationship and underlying mechanisms between secondhand smoke exposure and AF.

### The Association Between Alcohol Consumption and AF

The relationship between alcohol consumption and AF has been a subject of debate in prior research. A multitude of observational studies have documented a positive correlation between chronic heavy alcohol intake and the risk of AF [16, 47, 50], potentially attributable to alcohol induced myocardial fibrosis and alterations in atrial electrical activity [18, 35, 43]. However, O’Keefe et al. have proposed that moderate alcohol consumption exerts a protective effect on cardiovascular health [28] and have described a “J-shaped” curve relationship between alcohol consumption and the risk of cardiovascular diseases [39]. In our MR analysis, we did not detect any genetic susceptibility linking alcohol use, frequency of alcohol intake, and AF. We hypothesize that the effect of alcohol consumption on individuals may vary due to factors such as age [23], gender [55], genetic predispositions [36], and polymorphisms in enzymes involved in alcohol metabolism [45]. In this MR analysis, no direct causal relationship was identified between alcohol consumption and the risk of AF. Future real-world studies are crucial to further elucidate the relationship between alcohol consumption and AF.

This is the first bidirectional MR study elucidate the causal relationship between active smoking and AF, which is unaffected by confounding factors or reverse causality. The most significant strength of this research is its MR design, which bolsters causal inference. Additionally, the utilization of the most rigorous GWAS dataset substantially enhances causal validity. The study’s sample cohort, consisting solely of individuals of European descent, minimizes the bias stemming from population stratification. However, our research has several limitations. Firstly, given the GWAS participants are predominantly of European ancestry, direct extrapolation to other ethnic groups is not warranted due to racial diversity. Secondly, we detected heterogeneity in the analysis of current tobacco smoking and ever-smoked status. This heterogeneity might originate from diverse genetic backgrounds, the complexity of smoking behaviors, and potential confounding factors. To tackle this issue, we utilized the IVW random-effects model and conducted MR-PRESSO analysis to ensure the reliability of our findings. The results showed no significant alternations, suggesting that our findings were not affected by outliers. We acknowledge that despite employing multiple methods to control heterogeneity and potential confounding factors, unidentified confounders might still exist. Therefore, further studies with larger sample sizes are essential to replicate our findings in diverse racial populations and to investigate potential biological mechanisms and mediating factors between smoking and AF. Thirdly, despite the utilization of the highest-priority summary statistics, the availability of genetic instruments for smoking exposures, such as ever smoked, was very limited, leading to low statistical power. Further MR studies are necessary to validate these associations once more robust genetic tools become available.

## Conclusion

This study presents MR evidence supporting a causal association between active smoking and AF. The significance of smoking cessation is underscored by its potential to prevent or mitigate the risk of AF. The influence of secondhand smoke exposure and alcohol consumption on AF, as well as the causality among these factors, warrants further investigation.

## Data Availability

The original materials mentioned in the study can be obtained by contacting the corresponding author.

## Abbreviations

AF: Atrial fibrillation
MR: Mendelian randomization
GWAS: Genome-wide association study
IVW: Inverse-variance weighted
SNP: Single-nucleotide polymorphism
IVs: Instrumental variances
WM: Weighted median estimator
MR-PRESSO: Mendelian Randomization Pleiotropy Residual Sum and Outlier
OR: Odds ratio
CI: Confidence interval
